# Optimization of rVAR2-Based Isolation of Cancer Cells in Blood for Building a Robust Assay for Clinical Detection of Circulating Tumor Cells

**DOI:** 10.3390/ijms21072401

**Published:** 2020-03-31

**Authors:** Nicolai T. Sand, Tobias B. Petersen, Sara R. Bang-Christensen, Theresa D. Ahrens, Caroline Løppke, Amalie M. Jørgensen, Tobias Gustavsson, Swati Choudhary, Thor G. Theander, Ali Salanti, Mette Ø. Agerbæk

**Affiliations:** 1 Centre for Medical Parasitology at Department of Immunology and Microbiology, Faculty of Health and Medical Sciences, University of Copenhagen and Department of Infectious Diseases, Copenhagen University Hospital, 2200 Copenhagen, Denmark; nicolai@sund.ku.dk (N.T.S.); tobiaspetersen@sund.ku.dk (T.B.P.); sarabc@sund.ku.dk (S.R.B.-C.); theresa.ahrens@sund.ku.dk (T.D.A.); cabl@sund.ku.dk (C.L.); tobias@sund.ku.dk (T.G.); swati@sund.ku.dk (S.C.); thor@sund.ku.dk (T.G.T.); 2 VarCT Diagnostics, 2200 Copenhagen, Denmark; amj@varctdiagnostics.com

**Keywords:** Circulating tumor cells (CTCs), rVAR2, enrichment technologies, cancer, diagnostics, rare cell isolation

## Abstract

Early detection and monitoring of cancer progression is key to successful treatment. Therefore, much research is invested in developing technologies, enabling effective and valuable use of non-invasive liquid biopsies. This includes the detection and analysis of circulating tumor cells (CTCs) from blood samples. Recombinant malaria protein VAR2CSA (rVAR2) binds a unique chondroitin sulfate modification present on the vast majority of cancers and thereby holds promise as a near-universal tumor cell-targeting reagent to isolate CTCs from complex blood samples. This study describes a technical approach for optimizing the coupling of rVAR2 to magnetic beads and the development of a CTC isolation platform targeting a range of different cancer cell lines. We investigate both direct and indirect approaches for rVAR2-mediated bead retrieval of cancer cells and conclude that an indirect capture approach is most effective for rVAR2-based cancer cell retrieval.

## 1. Introduction

Early diagnosis of cancer drastically increases the chance of a successful treatment outcome [1,2,3]. Thus, major efforts are invested into developing high-throughput screening methods for individuals with an increased risk of developing cancer. Presently, most cancer screening programs such as mammography or colonoscopy target a specific organ. An alternative, non-invasive approach is the use of liquid biopsies that enable isolation of circulating tumor cells (CTCs), exosomes, or free circulating tumor DNA (ctDNA) from blood samples. CTCs are cells that have detached from the tumor site and entered the blood or lymphatic circulation. The dissemination of CTCs can lead to the formation of distant metastases resulting in cancer spread and poor prognosis. Importantly, many reports showed that levels of CTCs correlated with tumor progression along with the response to treatment and overall survival [4,5,6,7]. However, data suggest that CTCs are already shed from premalignant lesions, opening the possibility of using CTC detection for early diagnosis of cancer [8,9,10]. In addition, isolation of CTCs for phenotypic and genotypic analysis could provide a better understanding of tumor biology that is critical for disease monitoring and personalized treatment strategies. Therefore, liquid biopsies could be an appealing alternative to complicated, costly and painful tissue biopsies. However, most CTC isolation methods struggle to reach the necessary sensitivity for the technical challenge of detecting a few malignant cells among billions of normal blood cells.

We have previously demonstrated the use of chondroitin sulfate (CS) as a novel CTC marker [11,12]. Chondroitin sulfates are glycosaminoglycans consisting of long chains of repeated disaccharide units displayed on proteoglycans. Normally, CS is expressed by a wide variety of tissues [13], but a specific type of CS is expressed by the placenta and its dissimilarity from other types of CS is believed to originate in a distinct 4-*O* sulfation pattern [14,15]. Placental CS is the ligand for *Plasmodium falciparum*-infected erythrocytes (IE) binding in the intervillous space of the placental tissue [16]. The IE sequestration is mediated by the malaria-encoded VAR2CSA protein, which is imbedded on the erythrocyte cell membrane and binds placental CS with nanomolar affinity and high specificity [17,18]. Importantly, VAR2CSA does not mediate binding of IE to CS expressed elsewhere in the vasculature [19,20].

The VAR2CSA malaria protein is a large (350 kDa) multi-domain molecule. The minimal CS binding region is located to a 70 kDa subfragment in the N-terminal region spanning the DBL2 domain and the two interdomain regions designated ID1 and ID2 [19]. In 2015, it was shown that a smaller recombinant VAR2CSA protein encompassing ID1-ID2a (rVAR2) in addition to binding placental CS also bound to CS expressed by a vast majority of mesenchymal, epithelial and hematopoietic tumors [14]. This discovery led to terming the VAR2CSA ligand as oncofetal chondroitin sulfate (ofCS) [21].

Chondroitin sulfate proteoglycans have been associated with rapid cell proliferation as well as angiogenic and invasive properties, resembling key hallmarks of cancer [22,23]. Studies have demonstrated that ofCS has an essential role for tumor cell motility and invasion, and that rVAR2 effectively targets tumors in vivo [14,24,25]. We have previously shown that rVAR2-coated paramagnetic beads can isolate cancer cells spiked into blood as well as CTCs from blood samples of carcinoma and glioma patients [12,26]. To date, most immunomagnetic CTC enrichment platforms are designed to capture CTCs that express epithelial markers such as EpCAM. These techniques harbor a natural bias, as some epithelial cancers may undergo epithelial-to-mesenchymal transition and down regulate expression of EpCAM [27,28]. Thus, rVAR2 holds promise as an effective tool for isolating CTCs from a wide range of cancer entities.

Multiple CTC-capturing methods have been developed and show promising clinical results, however few publications have brought insights into the technical optimization and validation of these methods. In this study, we analyze the technical aspects of using rVAR2 as a cancer cell capture reagent in combination with magnetic beads. We assess the saturation of rVAR2 on magnetic beads by testing several rVAR2 to bead ratios and analyzing how these ratios affect cancer cell capture in buffer versus whole blood. Furthermore, we compare direct versus indirect capture of cancer cells and report high assay sensitivity as well as capture efficiency across various cancer cell lines. Our main aim has been to optimize these technical parameters to obtain the highest level of assay performance and reproducibility. In addition, we anticipate our findings may serve as an inspiration for the development and optimization of other bead-based CTC capturing technologies.

## 2. Results

### 2.1. Recombinant VAR2CSA can be Efficiently Coupled to Sera-Mag Beads

**Figure 1:**
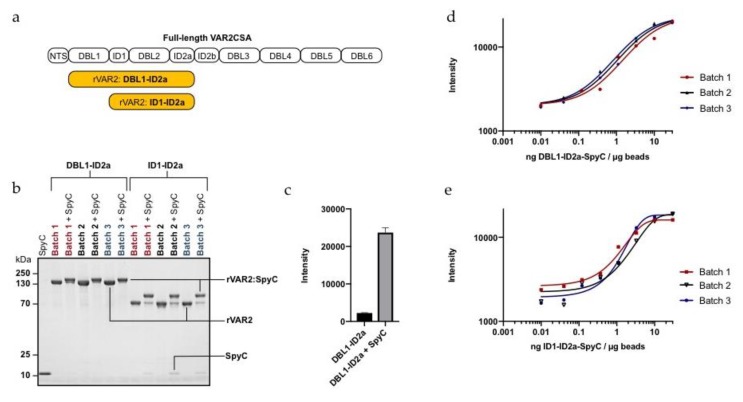
rVAR2-SpyC-coating of magnetic particles. (**a**) Schematic figure showing the domain structure of full-length VAR2CSA and the two recombinant subfragments (DBL1-ID2a and ID1-ID2a) used in this study (rVAR2, yellow). (**b**) SDS-PAGE showing the size-shift induced by coupling of biotinylated SpyC with different batches of SpyTagged DBL1-ID2a or ID1-ID2a. (**c**) Fluorescence intensity of anti-V5 FITC detection of DBL1-ID2a binding to Sera-Mag beads (30 ng rVAR2 / µg beads) showing rVAR2 bead binding dependency on coupling to biotinylated SpyC. Columns represent means of triplicates and error bars show SD. (**d**) Fluorescence intensity of anti-V5 FITC signal plotted against various rVAR2-SpyC to bead ratios of three different DBL1-ID2a and (**e**) ID1-ID2a batches.

Our initial CTC studies have shown promise for an rVAR2-based capture method, yet several technical aspects of the method remain unexplored. Previous clinical CTC data was based on the DBL1-ID2a (121 kDa) rVAR2 construct, but for this optimization study we also included the smaller ID1-ID2a (70 kDa) construct (Figure 1a) [26]. Both recombinant forms of rVAR2 are well defined in terms of ofCS specificity and affinity [19]. To control the protein quality, different production batches of the proteins were characterized for in vitro binding to chondroitin sulfate (CS) in ELISA. Both constructs of rVAR2 bound specifically to CS-carrying decorin while there was negligible binding towards heparan sulfate proteoglycan (HSPG) (Figure S1). The binding affinity was additionally tested by rVAR2 binding to MyLa 2059 cancer cells. Flow cytometry showed a concentration-dependent binding as well as complete inhibition of binding by addition of soluble CSA affirming the specificity of rVAR2 binding to CS (Figure S2).

Many commercially available beads for cell capture are based on the high affinity interaction between biotin and streptavidin. When using streptavidin-coated beads, a biotinylated form of the capture reagent is required. Although biotin is only a 0.2 kDa-sized molecule, direct coupling of multiple biotins to rVAR2 could potentially interfere with the protein functionality or affect the protein orientation when coupled to the beads. Instead, rVAR2 coupling to the beads was mediated through the split intein (SpyTag/SpyCatcher) conjugation system (Figure S3) [29]. Both DBL1-ID2a and ID1-ID2a proteins were expressed with a genetically fused SpyTag. When mixing SpyTagged rVAR2 with a biotinylated recombinant SpyCatcher (SpyC), an irreversible isopeptide bond is formed between the SpyTag and SpyC leading to biotinylation of rVAR2. Successful conjugation of the 13 kDa SpyC to three different batches of each SpyTagged rVAR2 proteins was confirmed by SDS page (Figure 1b).

The biotinylated rVAR2-SpyC complexes were conjugated to Sera-Mag^TM^ SpeedBeads Streptavidin-Blocked Magnetic Particles (Sera-Mag beads), which are 1 µm streptavidin-coated beads with a polystyrene core and double-layered magnetite [30]. rVAR2 binding to the beads was quantified by exploiting a V5-tag present at the C-terminus of the rVAR2 proteins. The rVAR2-coated beads were stained with anti-V5 FITC antibody. Importantly, no non-specific binding of rVAR2 to the beads was detected in the absence of the biotinylated SpyC (Figure 1c). For both DBL1-ID2a and ID1-ID2a, saturation was obtained at ~10 ng rVAR2-SpyC per µg beads (Figure 1d,e).

### 2.2. rVAR2-Coating of Beads is Required for Capture of Cancer cells

**Figure 2:**
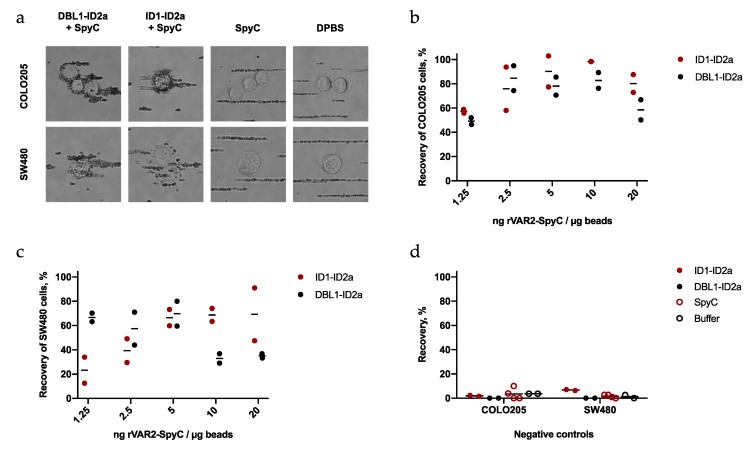
Bead binding to cancer cells in buffer is dependent on rVAR2-SpyC-coating. (**a**) Representative bright field images of COLO205 and SW480 cells incubated with beads coated with DBL1-ID2a-SpyC, ID1-ID2a-SpyC, SpyC, or DPBS. Images are taken with 40× objective, CellCelector (Automated Lab Solutions). (**b**) and (**c**) Recovery of COLO205 or SW480 cells from PF buffer using various amounts of rVAR2-SpyC per µg beads. Lines represent mean values. (**d**) Capture efficiency for the negative control beads prepared with non-biotinylated rVAR2, SpyC, or buffer. Lines represent mean values of duplicates or quadruplicates.

To show that bead binding to cancer cells is dependent on rVAR2-conjugation, two colorectal cancer cell lines (COLO205 and SW480) were incubated with beads coated with DBL1-ID2a-SpyC or ID1-ID2a-SpyC. Additionally, naked beads and beads only mixed with SpyC were used as negative controls. As expected, naked beads and SpyC-coated beads exhibited none or very little binding to the cancer cells (Figure 2a). However, when adding biotinylated rVAR2-SpyC to the beads, binding of the beads to cancer cells was observed for both rVAR2 constructs.

To determine the optimal concentration of rVAR2-SpyC on the beads, we tested the effect of different protein to bead ratios. Assay efficacy was measured by the recovery of 100 cancer cells spiked into protein-free (PF) buffer. The two colorectal cancer cells lines, COLO205 and SW480, were pre-stained with CellTracker^TM^ Green (CTG) and incubated with rVAR2-coated Sera-Mag beads. The bead-bound cells were then isolated using a magnet, fixed, and stained with DAPI to enable visualization of cell nuclei. CTG^+^/DAPI^+^ cells were enumerated using a Cytation 3 microscopy scanner. Consistently for both ID1-ID2a and DBL1-ID1a, the highest recovery of cancer cells was observed when using 2.5–10 ng rVAR2-SpyC per µg beads (Figure 2b,c). Importantly, the capture of the cancer cells was dependent on the combination of rVAR2 and biotinylated SpyC as no or very little recovery was achieved when using either one alone (Figure 2d).

### 2.3. rVAR2 Captures Cancer Cells from Blood

**Figure 3:**
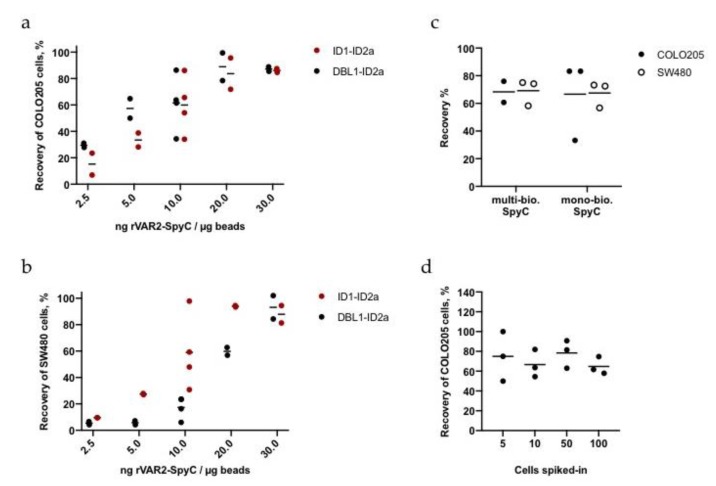
Optimization of the rVAR2 to bead ratio for capture of cancer cells in blood. (**a**) and (**b**) Recovery of COLO205 or SW480 cells from 1 mL blood after capture experiments with different protein to bead ratios. Each ratio was tested in duplicates or quadruplicates. Lines represent mean values. (**c**) Capture experiment of COLO205 and SW480 cells spiked into blood using DBL1-ID2a with either multi- or mono-biotinylated SpyCatcher linkage to magnetic beads. Lines represent mean values. (**d**) Sensitivity measurements of the direct capture method of approximately 5, 10, 50, or 100 COLO205 cells in 3 mL blood using DBL1-ID2a. Lines represent mean values. The exact cell numbers can be found in Table S1.

Although an rVAR2 concentration of 10 ng/µg beads saturated the Sera-Mag beads and 5 ng/µg led to a good recovery of cancer cells from buffer, the optimal protein to bead ratio was re-evaluated for capturing cancer cells spiked into whole blood. COLO205 or SW480 cells were spiked into 1 mL blood samples with 100 cancer cells per sample (Figure S3). Following RBC lysis, rVAR2-coated Sera-Mag beads were added to the cell samples. Surprisingly, an oversaturated protein to bead ratio (30 ng/ug) yielded the highest capture efficiency for both cancer cell lines (Figure 3a,b). The use of even higher rVAR2-SpyC concentrations on the beads has not been tested in cell capture experiments, as it resulted in bead aggregation when using both protein-free (PF) or bovine serum albumin (BSA)-based coupling buffers (Figure S4).

The aggregation of beads at a high rVAR2 concentration may be caused by the use of a multi-biotinylated SpyC, increasing the risk of several streptavidin molecules interacting with the same SpyC. Since the capture of cancer cells from blood was most optimal using oversaturating amounts of rVAR2, a mono-biotinylated SpyC was also tested for bead binding efficacy and cancer cell recovery. Bead binding efficacies, bead aggregation, and cancer cell recoveries using either mono- or multi-biotinylated SpyC were however similar (Figure 3c and Figure S5a). Thus, for all further capture experiments we used 30 ng DBL1-ID2a-SpyC per µg beads and a multi-biotinylated SpyC.

Next, we tested the sensitivity of the assay by spiking a dilution of cancer cells into blood. COLO205 cells were pre-stained with CTG and a predetermined number of cells ranging from 5 to 100 were spiked into 3 mL blood. The samples were processed as described in Materials and Methods. Notably, the assay efficiency in 3 mL blood was maintained at the low cell concentrations, with mean recoveries of 65–78% when spiking in ~5 cells (Figure 3d and Table S1).

### 2.4. rVAR2 Binds to and Captures a Variety of Cancer Cells when Coupled to Magnetic Beads

**Figure 4:**
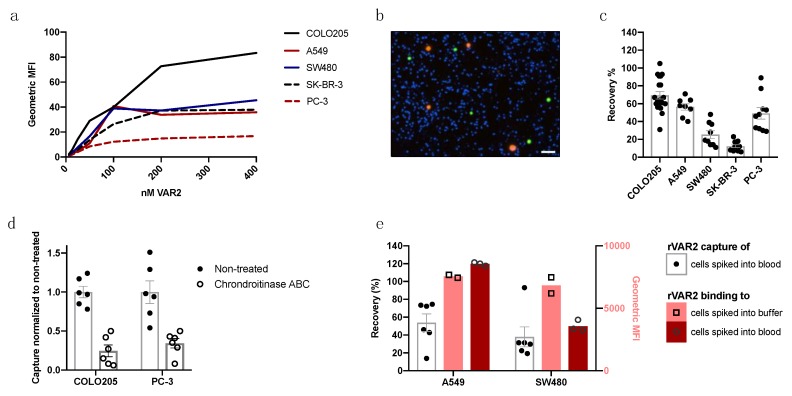
rVAR2 binds to and captures a variety of cancer cell lines when coupled to magnetic beads. (**a**) Flow cytometry shows rVAR2 binding to five cancer cell lines in buffer determined by anti-V5 FITC antibody geometric mean fluorescence intensity. Results are presented as mean of duplicates. (**b**) Representative image of captured COLO205 cells (green) and A549 cells (orange) in a background of DAPI+ white blood cells (blue). Image is taken with a 4× objective, Cytation 3. Scale bar 50 μm. (**c**) rVAR2-based recovery of the five different cell lines COLO205 (*n* = 20), A549 (*n* = 8), SW480 (*n* = 9), SK-BR-3 (*n* = 12) and PC-3 (*n* = 10) from 3 mL blood samples. Each dot represents a sample recovery and error bars show +/- SEM. (**d**) Recovery of COLO205 and PC-3 with or without chondroitinase ABC pre-treatment. Chondroitinase ABC-treated samples were normalized to the mean of the recovery for the non-treated samples. Each dot represents a sample recovery and error bars show +/- SEM. (**e**) Parallel experiment on cell-matched samples on rVAR2-based capture of 100 CTO^+^ A549 or SW480 cancer cells in 3 mL of blood (black) and test of 200 nM rVAR2 binding to the CTO^+^ cancer cells in buffer (pink) or spiked into blood and RBC-lysed (red). rVAR2 binding was measured by anti-V5 FITC staining in flow cytometry (MFI, mean fluorescence intensity). Columns represent mean values and error bars show +/- SEM.

After confirming a highly sensitive capture of COLO205 cells from 3 mL blood samples, we expanded the testing to a total of five cancer cell lines of different tumor origin (COLO205, A549, SW480, SK-BR-3, and PC-3). First, rVAR2 (DBL1-ID2a) binding to the cancer cells in buffer was quantified by flow cytometry (Figure 4a). The maximum binding capacity of rVAR2 varied across cell lines, which suggested differences in ofCS expression, display or accessibility.

Subsequently, the panel of different cancer cell lines was used in spike-in experiments to test the capture efficiency of the assay. One hundred cancer cells were pre-stained with CTG or CellTracker^TM^ Orange (CTO) and used in spike-in experiments to test the capture efficiency from 3 mL blood. An example of a Cytation 3-scanned image of recovered COLO205 and A549 cells spiked into the same blood sample is shown in Figure 4b. rVAR2-based isolation led to a decent recovery of the COLO205, A549, and PC3 cells (69.4%, 56.4%, and 49.1%, respectively), whereas the SW480 and SK-BR-3 cells were poorly recovered from 3 mL blood samples (25.3% and 12.3%, respectively) (Figure 4c). This was surprising, as rVAR2 binding by flow cytometry in buffer did not suggest this outcome (Figure 4a).

In order to verify the CS-specificity of the interaction between rVAR2-conjugated beads and cancer cells, rVAR2 capture of cancer cell lines was assessed with or without a pre-treatment with chondroitinase ABC. Common for both the high rVAR2-binding COLO205 cells and the lower rVAR2-binding PC-3 cells was a significant decrease of capture efficiency when cells were treated with chondroitinase ABC prior to spike-in (Figure 4d).

In order to further investigate the discordance between rVAR2 binding to cancer cells and rVAR2-mediated capture of the cancer cells from blood, we ran both assays in parallel. For this, the cell lines A549 and SW480 were selected, because both cell lines showed similar rVAR2 binding in buffer (Figure 4a), but showed differences in capture efficiency (56.4% for A549, but only 25.3% for SW480, Figure 4c). We therefore investigated binding to these cancer cell lines in both buffer and blood in parallel with capture to investigate whether rVAR2 binding to the cancer cells was affected upon spike-in to blood. Cells grown in the same culture flask were used for both the flow cytometry and capture assay to rule out differences in cell culture condition and handling. Interestingly, rVAR2 binding to A549 cells in buffer versus blood did not differ, while binding to SW480 cells dropped dramatically when the cells had been suspended in blood, which could explain the decreased recovery rate of the SW480 cells (Figure 4e).

### 2.5. An Indirect Capture Approach Increases the Recovery of Cancer Cell Lines

**Figure 5:**
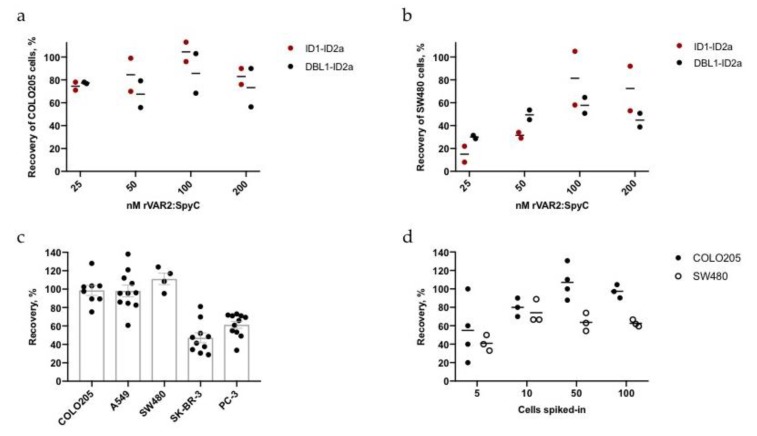
rVAR2-based indirect capture of cancer cells. (**a**) and (**b**) Recovery of 100 COLO205 or SW480 cells spiked into 3 mL blood using 25–200 nM biotinylated rVAR2-SpyC prior to bead incubation. Lines represent mean values of duplicates. (**c**) Recovery of 100 cells for each of the five cancer cell lines COLO205 (*n* = 8), A549 (*n* = 10), SW480 (*n* = 4), SK-BR-3 (*n* = 10) and PC-3 (*n* = 11) from 3 mL blood samples using 200 nM biotinylated DBL1-ID2a-SpyC prior to bead incubation. Each dot represents a sample recovery and error bars show +/- SEM. (**d**) Sensitivity testing of the indirect capture approach using 200 nM biotinylated DBL1-ID2a-SpyC for the retrieval of approximately 5–100 COLO205 or SW480 cells spiked into 3 mL blood. The exact cell number can be found in Table S3.

Two strategies may be applied for magnetic isolation of target cells in a complex sample: A direct capture method, where the capture reagent is immobilized onto the beads prior to encounter with the cell sample, or an indirect capture method, where cell samples are first incubated with the capture molecule and then incubated with the beads. So far, the direct capture method facilitated a highly sensitive capture of COLO205 cells but resulted in varying capture efficiency of other cell lines, such as SW480 or SK-BR-3. Since all cell lines bound rVAR2 as measured by flow cytometry, we tested whether the capture efficiency could be improved by utilizing an indirect capture approach, where cells are incubated with biotinylated rVAR2-SpyC prior to adding the beads. Various concentrations of biotinylated ID1-ID2a-SpyC or DBL1-ID2a-SpyC were incubated with cancer cells after spike-in to 1 mL blood samples. Recovery of the COLO205 cells was not much affected by the change in rVAR2-SpyC concentration (Figure 5a). However, when testing the lower rVAR2-binding SW480 cell line, the capture efficiency was highest using 100–200 nM rVAR2 (Figure 5b).

Next, the recovery of the five cancer cell lines (COLO205, A549, SW480, SK-BR-3, and PC-3) was re-assessed using 200 nM biotinylated rVAR2 in the indirect approach for capture of approximately 100 cancer cells from 3 mL blood (Figure 5c). Interestingly, all cell lines except PC-3 showed significantly increased capture rates when using the indirect approach compared to the direct approach (Table S2). For example, the mean recovery of the SW480 cells increased from 25% to >100%. Similarly, the mean recovery of A549 cells increased from 56% to 98%. For each spike-in experiment, the mean of three validation counts of the CellTracker^TM^-stained cancer cells was used to determine the 100% reference point. Recovery rates exceeding 100% are most likely caused by the variation of the technical spike-ins.

We then tested the sensitivity of the indirect method. A series of capture experiments with a dilution of COLO205 and SW480 cells in 3 mL blood was performed. With the indirect capture approach, we achieved a highly sensitive capture for both the high rVAR2-binding COLO205 cells and the lower rVAR2-binding SW480 cells (Figure 5d and Table S3). For a comparison between sensitivity of the direct and indirect approaches see Tables S1 and S3.

## 3. Discussion

Capture of rare CTCs from clinical blood samples requires an assay that is efficient, highly sensitive and robust. Targeting oncofetal chondroitin sulfate (ofCS) by the malarial recombinant VAR2CSA (rVAR2) protein might hold potential as a near-universal CTC-targeting agent as it binds to cancer cells independently of tumor origin. Furthermore, we have previously shown that rVAR2 binding to cancer cells is unaffected by cell plasticity such as epithelial-to-mesenchymal transition (EMT) [26]. This is an important trait since EMT is indicative of increased metastatic capacity of CTCs [31]. Thus, a capture technique, which is independent of epithelial markers, such as EpCAM, would be desirable to target a broad range of cell states or cancer entities. This study therefore set out to explore and optimize different technical parameters and approaches for rVAR2-based cancer cell isolation. We have previously shown that rVAR2-coated paramagnetic beads can isolate CTCs from clinical cancer patient blood samples [12,26]. However, in this study, the aim was to further optimize the technical parameters to obtain the highest level of assay performance and the required reproducibility, as well as paving the way for a standardized optimization method of bead coupling to rVAR2, which could also inspire development strategies of other bead-coupled CTC-targeting moiety technologies. The rVAR2-based method may ultimately be used in a clinical CTC capturing setting to diagnose and monitor disease progression in a wide range of cancer types.

In this study, we evaluated the effect of using two different subfragments of rVAR2, DBL1-ID2a and ID1-ID2a, for cancer cell isolation. As we did not observe consistent superiority of either of the constructs in regards to cancer cell capture, we continued with the DBL1-ID2a construct as our standard construct for any proceeding experiments.

As biotinylation and direct bead coupling of rVAR2 could affect the ofCS binding capacity, we used a non-disrupting technology for coupling the protein to beads. The SpyTag/SpyCatcher system provides an effective plug-and-play tool for conjugating magnetic beads with a cancer cell-targeting moiety. The SpyTagged rVAR2 proteins were efficiently coupled to biotinylated SpyC, leaving little free SpyC in the protein mix to occupy streptavidin residues on the magnetic beads.

Many commercially available beads for cell capture are based on the high affinity interaction between biotin and streptavidin. In this study, we used the Sera-Mag^TM^ SpeedBeads Streptavidin-Blocked Magnetic Particles. These beads were chosen based on their high biotin binding capacity and low dissociation constant. Furthermore, in contrast to the CELLection^TM^ Dynabeads^®^ previously employed in our studies, the Sera-Mag^TM^ SpeedBeads exhibit low non-specific protein binding and low bead autofluorescence facilitating fluorescence microscopy analysis of captured cancer cells [30]. Throughout this study, we demonstrated that rVAR2-coating of the beads was dependent on an efficient biotinylation of rVAR2. In addition, non-rVAR2-coated beads showed no or low (<5%) capture of cancer cells. Together, this data indicates that the surface of Sera-Mag beads ensures minimal interaction with non-biotinylated reagents enabling specific targeting of cancer cells through the rVAR2 protein.

Density curves of rVAR2-SpyC complex binding to the Sera-Mag beads showed a saturation around 10 ng/µg. This value is comparable to a previous study testing anti-EpCAM antibody binding to Sera-Mag beads [30]. High protein to bead ratios (100 ng rVAR2-SpyC per µg beads) in the buffer resulted in bead aggregation. We used a multi-biotinylated SpyC, which may have bound to multiple streptavidin units leading to formation of bead complexes. For that reason, we tested the influence of using mono- versus multi-biotinylated SpyC on bead binding. However, the rVAR2 density curves showed no pronounced variance nor did we observe any change in cancer cell capture.

Besides the potential risk of bead clumping, we speculated that an oversaturation of the beads could be unfavorable as a high-density display might cause steric hindrance obstructing the capturing capacity. In line with this, an oversaturated protein to bead ratio (>10 ng rVAR2-SpyC per µg beads) led to a slightly decreased recovery efficiency of cells spiked into buffer. However when applying the protocol to blood samples, an oversaturated protein to bead ratio of 30 ng/µg yielded optimal recoveries. In theory, any additional rVAR2 above 10 ng/µg beads should be removed during the preparation and wash of the beads. Any residual free rVAR2 in the bead preparation could however potentially affect the cell recovery when added to the cell sample. As the added benefit of oversaturated beads is only observed in blood spike-ins, it could be speculated that free rVAR2 has a blocking effect on residual plasma components or WBCs. Further studies are however required for more insights into this phenomenon. More importantly, protein to bead ratio determined based on capture of cancer cells in buffer was not comparable to capture from blood samples, demonstrating why optimization should always be performed on samples mimicking true cancer patient blood samples as closely as possible.

When testing the direct capture approach on five different cancer cell lines, it became clear that the rVAR2-based capture from blood samples varied substantially among these cell lines. We speculated that differences in ofCS expression, display, or accessibility could potentially account for these differences. However, the difference in recovery between the cell lines could not be explained or correlated to the variations in rVAR2 binding to cancer cells as measured by flow cytometry in buffer. Yet, the accessibility of ofCS on cancer cells could have been affected after the spike-in to blood. In order to investigate a proper correlation between rVAR2 binding to cancer cells and spike-in capture efficiency, we therefore performed simultaneous flow cytometry and capture experiments on A549 and SW480 cells spiked into blood. rVAR2 binding to SW480 cells from buffer and blood samples differed remarkably, which could explain the low recovery rate of this cell line in blood. In contrast, rVAR2 binding to A549 cells appeared unaffected by the blood component, corresponding to the high recovery rate. In this view, the cell lines SW480 and SK-BR-3, which had a poor recovery when using the direct capture method, might be susceptible to alterations in ofCS accessibility when spiked into blood, while COLO205, A549 and PC-3 cells appear relatively unaffected. These findings further underline the importance of studying cancer cells spiked into blood when developing CTC-capturing technologies. As ofCS is displayed on a variety of CSPGs, which are differently expressed by different cancer cell lines, the reason for capture efficiency deviations may be complex. Some CSPGs may be more affected by sample processing, interact with blood components, or be more hidden in the cancer cell glycocalyx, while other CSPGs remain available to rVAR2 binding.

Although direct CTC capture methods based on pre-coated magnetic beads is widely applied, an indirect strategy based on pre-incubation of the sample with a CTC-targeting moiety prior to bead conjugation may yield increased sensitivity in the case of low target expression [30]. Only a few studies describing CTC capture technologies apply both of these methods in a comparative setup [30,32]. When we applied the indirect method, we obtained a considerable increase in the recovery rates of the cancer cell lines compared to the direct method. This may indicate that free rVAR2 has better access to its target ofCS compared to rVAR2 immobilized on magnetic beads, leading to a more efficient capture. Most importantly, the increased recovery of SW480 cells by indirect capture was maintained even when spiking as few as 5 cells into 3 mL blood samples, demonstrating the high degree of specificity towards this cell line. Combined with the finding that SW480 cells show a reduction in rVAR2 binding after incubation in whole blood, this data suggests that even if circulating tumor cells from patient samples are binding relatively low levels of rVAR2, this optimized assay would be suitable for their detection.

Although this study attempted to encompass the optimal performance of the two rVAR2 proteins for capture of cancer cells, there are still several parameters, which could be explored and optimized such as buffers, timing, automatic handling and testing of different bead types. In this study, the Sera-Mag beads proved low non-specific binding, and we observed negligible property differences when comparing the performance of the two rVAR2 constructs. The most dramatic improvement in recovery rates was obtained when implementing the indirect approach. The indirect method involves more handling and incubation time and as CTCs are per definition not found in their natural habitat and therefore suffer environmental and physical stress, time is of the essence when capturing CTCs. Yet, the indirect method greatly outcompetes the direct method in this initial technical test. Clearly, investigations on capture rate of CTCs from clinical samples will be required to understand the true impact of this improved rVAR2-based cancer cell isolation approach. We believe that this optimization study is an important step to build a clinical rVAR2-based diagnostic assay for CTC detection. Furthermore, we hope that our workflow for parameter optimization might serve as inspiration for the development of other magnetic bead- and antibody-based CTC-capturing technologies.

## 4. Materials and Methods

### 4.1. Protein Production and Quality Testing

VAR2CSA was produced in two recombinant versions (rVAR2), DBL1-ID2a and ID1-ID2a, in SHuffle® T7 Competent *E. coli* as previously described [12]. Both constructs include a C-terminal hexa His-tag and V5 tag, as well as an N-terminal SpyTag. Proteins were purified to homogeneity and the different batches of rVAR2 were characterized for specific binding towards CSA using flow cytometry and ELISA.

For binding ELISA, a 96 well microtiter plate (351172, BD Life Sciences, Franklyn Lakes, USA) was coated with 3 μg/mL decorin (D8428, Sigma-Aldrich, St. Louis, USA) or HSPG (H4777, Sigma-Aldrich) overnight at 4 °C. Plates were blocked using TSM buffer (20 mM Tris, 150 mM NaCl, 2 mM CaCl_2_, 2 mM MgCl_2_, 0.05% Tween 20, pH 7.4) containing 1% BSA followed by incubation with a 2-fold dilutions of rVAR2 in the same buffer (100-1.56 nM, 1 h at 37 °C). The plates were washed thrice in TSM buffer, and the bound rVAR2 was detected using anti-His-HRP (C-term.) antibody (1:3000, 130-092-783, Miltenyi Biotec, Bergisch Gladbach, Germany). Absorbance was measured at 490 nm using an ELISA reader after development with OPD tablets (4110H, Kem-En-Tec, Nordic A/S, Taastrup, Denmark) for 12 min and reaction quenching by 1 M H_2_SO_4_.

In vitro binding was tested on MyLa 2059 cells. 200,000 MyLa 2059 cells were incubated with a 2-fold dilution of rVAR2 (400-6.25 nM) or 200 nM rVAR2 with 400 ug/mL CSA (27042, Sigma). rVAR2 binding was detected using Penta-His Alexa Fluor 488 Conjugate (1:200, 35310, Qiagen, Hilden, Germany) using flow cytometry as described in Section 4.7.

SpyCatcher (SpyC) was produced in the *E. coli* BL21 strain and multibiotinylated using NHS biotin as previously described [12]. Furthermore, the SpyCatcher was mutated to carry a cysteine at a single position (S8C) and was similarly produced in *E. coli* BL21 strain. SpyCatcher S8C was mono-biotinylated using the maleimide-biotin (B1267, Sigma-Aldrich).

### 4.2. Preparation of rVAR2-Coated Sera-Mag Beads

rVAR2 and the biotinylated SpyC were mixed in a 1.2:1 ratio (see test of various ratios in Figure S5b) and left for 1 h at room temperature to form a covalent isopeptide bond via the SpyTag-SpyC interaction as described previously [29]. Sera-Mag^TM^ SpeedBeads Streptavidin-Blocked Magnetic Particles (21152104010150, GE Healthcare, Little Chalfont, UK) solution was vortexed and added to a low-bind microcentrifuge tube and washed twice in 1 mL Pierce^TM^ Protein-Free (PBS) Blocking Buffer (PF buffer) (37572, Thermo Fisher Scientific, Waltham, USA). The beads were resuspended in PF buffer to a final concentration of 1.67 µg beads/µL. Whenever beads were used for the indirect capture method, beads were added directly to the cell sample from this step. For rVAR2-coating of the beads, the rVAR:SpyC protein mix was added to the bead solution, mixed with the magnet, and left to conjugate at room temperature for 1 h. Lastly, the beads were washed twice to remove unbound protein and resuspended in PF buffer to reach final concentration of 1.67 µg beads/µL.

### 4.3. Determination of rVAR2 Density on the Magnetic Particles

Determination of the saturation point for the binding of rVAR2 to beads involved the preparation of the beads as described in Section 4.2. Different production batches of rVAR2 were incubated with SpyC and added to the beads in concentrations ranging from 0.01 to 100 ng rVAR2-SpyC per μg beads.

Beads were then washed twice in BSA-based buffer (0.1% BSA, 2 mM EDTA in DPBS) prior to adding anti-V5 FITC antibody solution (R963-25, Invitrogen, Waltham, USA) diluted in BSA-based buffer. After incubation at room temperature for 30 min, the beads were washed twice in Dulbecco’s Phosphate Buffered Saline modified without Ca^2+^ and Mg^2+^ (DPBS) (D8537, Sigma), fixed in 4% paraformaldehyde (PFA) (J61899.AK, Alfa Aesar, Haverhill, USA) for 5 min and resuspended in DPBS containing 10% PF buffer.

Each sample was transferred to the middle of the well in a 24-well glass bottom Sensoplate^TM^ (662892, Greiner Bio-One, Kremsmünster, Austria). A magnet located directly underneath the individual wells enabled the transfer and mounting using Faramount Aqueous Mounting Media (DAKO). The plate was scanned using the Cytation^TM^ 3 Cell Imaging Multi-Mode Reader. The samples were analyzed using a 20× objective and the GFP filter (BT1225101, EX 469 nm, EM 525 nm, BioTek Instruments, Inc., Winooski, USA), with an LED intensity setting of 7, integration time 600 and gain 10. The data was analyzed using Gen5^TM^ Microplate Reader and Imager Software (BioTek Instruments, Inc.) and background intensity of the FITC signal emitted from the beads was plotted against the concentration of rVAR2-SpyC to determine the saturation point.

### 4.4. SDS-PAGE

The rVAR2 proteins were tested by SDS-PAGE to confirm purity and SpyC conjugation ability. rVAR2 was mixed with SpyC in a 1:1 ratio for 30 min. The mixture was then diluted in DPBS to reach a rVAR2 concentration of 0.1 µg/µL. 12.5 µL rVAR2-SpyC conjugation mixture was prepared with 2.5 µL SDS (+/− DTT) solution. The samples were placed in a 95 °C heating block for 10 min prior to running them on a NuPAGE 4–12% Bis-Tris SDS-PAGE gel (Life technologies, NP0321PK2) with a PageRuler^TM^ Plus prestained protein ladder (10–250 kDa) (26619, Thermo Fisher Scientific) as a size reference. The gel was run at 170 V for 1 h and was subsequently stained with InstantBlue Coomassie Protein Stain (ISB1L, Expedeon, Cambridge, UK) for at least 1 h. The gel was destained in deionized water for 1 h prior to imaging.

### 4.5. Cell Culture

MyLa 2059, COLO205 and SW480 cells were cultured in RPMI 1640 medium with GlutaMax^TM^ supplement, while A549, PC-3 and SK-BR-3 cells were cultured in DMEM medium with GlutaMax^TM^ supplement. Both media were acquired from Sigma Aldrich and supplemented with 10% fetal bovine serum (FBS and 1% penicillin-streptomycin. The cell lines were regularly detached using Trypsin-EDTA Solution 1× (59417, Sigma-Aldrich) for passaging and sustained at 5% CO_2_ and 37 °C. The cells were passaged 24 or 48 h before experimental use.

### 4.6. Validation of Bead Binding Specificity

COLO205 or SW480 cells in RPMI 1640 medium with GlutaMax^TM^ supplement were incubated with beads that had been mixed with DBL1-ID2a:SpyC, ID1-ID2a:SpyC, DBL1-ID2a, ID1-ID2a, SpyC or DPBS. The samples were then placed on a glass slide using a magnetic adapter for bead immobilization and visualized on a 40× objective CellCelector^TM^ (Automated Lab Solutions GmbH, Jena, Germany).

### 4.7. Flow Cytometry

The binding of rVAR2 to cancer cell lines was confirmed by flow cytometry. Adherent cells were detached by CellStripper® Solution (25056CI, Corning®,) or StemPro^TM^ Accutase^TM^ Cell Dissociation Reagent (A1110501, Gibco, Waltham, USA). For experiments in buffer, approximately 200,000 cells were directly transferred to a U-bottom 96-well microplate (3799, Corning, Corning, USA). For experiments in blood, cancer cells were pre-stained with CellTracker^TM^ Orange CMRA Dye (C34551, Thermo Fisher Scientific) as described in Section 4.8 and 300,000 cells were spiked into 3 mL blood. The blood was process as described in Section 4.10. Cells were finally resuspended in DPBS + 2% FBS (PBS2) and transferred to a U-bottom 96-well microplate. The plate was centrifuged at 500× g for 5 min (4 °C) and incubated with two-fold serial dilution of rVAR2 (6.25–400 nM DBL1-ID2a) in DPBS2 and left shaking for 30 min at 4 °C. The samples were then washed twice in DPBS2 prior to incubation with anti-V5 FITC solution (1:500, R963-25, Invitrogen,) diluted in DPBS2 for 30 min at 4 °C. Finally, the samples were washed twice, resuspended in DPBS2 and processed on an FC500 flow cytometer (Beckman Coulter, Brea, USA) or an LSR II flow cytometer (BD^TM^). Data were analyzed using FlowJo^TM^ software (BD Life Sciences) and the results are presented as the geometric mean of the FITC signal. Two experiments including duplicate samples of COLO205, SW480, SK-BR-3 and A549 cells or COLO205, SW480 and PC-3 cells were merged into one graph.

### 4.8. Preparation of Cells for Spike-In

Adherent cancer cells were washed in DPBS and gently detached using CellStripper® Solution or StemPro^TM^ Accutase^TM^ Cell Dissociation Reagent. Both reagents preserve ofCS display on the cancer cells, yet Accutase more efficiently dissociates cell clumps and thereby facilitates uncomplicated validation counting. The suspension cell fraction of COLO205 cells was directly centrifuged and further processed. The cells were stained with CellTracker^TM^ Green CMFDA Dye (C7025, Thermo Fisher Scientific) or CellTracker^TM^ Orange CMRA Dye (C34551, Thermo Fisher Scientific) according to manufacturer’s instructions. After staining, cells were resuspended in complete media and allowed to recover at 37 °C for minimum 30 min. Viable cells were counted in a 1:1 mixture with 0.4% Trypan Blue (Sigma-Aldrich) using a haemocytometer. A cell suspension with concentration of 10,000 cells/mL was prepared in DPBS, and 10 µL (100 cells) was spiked into either blood or buffer as specified in the Results section. To validate the actual amount of cells spiked in, three 10 µL droplets of the DPBS cell suspension were transferred to a glass slides and cells were manually counted. The mean of these three counts was set to 100% in order to determine the recovery efficiency. For the sensitivity assay, further cell dilutions of 5000 and 1000 cells/mL (50 and 10 cells per 10 µL) were made. A 10 times dilution of the 5000 cells/mL solution was made and droplets of 10 µL were transferred to a flatbottom 96-well plate. The cells were manually counted and the droplets holding around 5 cells were spiked directly into the blood.

### 4.9. Chondroitinase Treatment

For chondroitinase ABC pre-treatment conditions, cells were incubated in DPBS2 supplemented with 0.25 mU/µL chondroitinase ABC from Proteus vulgaris (C2905-5UM, Sigma-Aldrich) at 37 °C for 30 min. This was done prior to counting and spike-in to blood.

### 4.10. Processing of Blood

Blood was drawn from healthy donors using K2E (EDTA) Vacutainer^®^ blood collection tubes (367525, BD) or LBgard^®^ Blood Tubes (BioMatrica, San Diego, USA) 0–5 h before the start of the experiment. Cells were spiked into either 1 or 3 mL of blood as specified in the Results section. Red blood cell lysis buffer was added to a final concentration of 0.155 M NH_4_Cl, 0.01 M KHCO_3_ and 0.1 mM EDTA and samples were incubated at room temperature for 13 min. Samples were then centrifuged at 400× *g* for 8 min, the pellet washed in 3 or 6 mL DPBS (when processing 1 or 3 mL blood, respectively), and centrifugation was repeated.

### 4.11. Cancer Cell Capture from Blood

For direct capture, the samples were dissolved in 250 or 600 µL cold PF buffer using a low-retention pipette tip. The samples were transferred to a 0.5 or 1.5 mL low-retention microcentrifuge tube (Fisher Scientific International, Inc., Pittsburgh, USA), which had been pre-coated with PF buffer and immediately put on ice. A 15 µL rVAR2-coated bead suspension (25 µg beads) was added to each sample. The samples were left rotating at 4 °C for 30 min to allow binding of the cells.

For the indirect capture, the samples were centrifuged and resuspended in DPBS with 5% FBS and 1 mM EDTA. rVAR2-SpyC was added to the cell suspension to obtain a final concentration of 25 to 200 nM and samples were incubated rotating for 30 min at 4 °C. Samples were then centrifuged for 8 min at 350× *g* and washed in 600 µL BSA-based buffer (0.1% BSA, 2 mM EDTA in DPBS) to remove excess rVAR2-SpyC. Following resuspension in 600 µl BSA-based buffer, washed 15 µL Sera-Mag beads (1.67 µg beads/µL) were added and the samples were left rotating at 4 °C for 30 min.

After cell:bead incubation, bead-bound cells were isolated using a magnet, fixed in 100 µL 4% PFA (J61899.AK, Alfa Aesar) for 5 min and resuspended in DPBS containing 10% PF buffer. For microscopic analysis, the cell nuclei were stained with DAPI (D1306, Life Technologies) in DPBS for 5 min at room temperature and finally resuspended in DPBS.

Each sample was transferred to a 24-well glass bottom Sensoplate^TM^ as described in Section 4.3. The plate was then scanned using a 4× objective of the Cytation^TM^ 3 Cell Imaging Multi-Mode Reader and manually analyzed for DAPI^+^ and CellTracker^+^ cells using the Gen5^TM^ software (BioTek), thereby determining the cancer cell recovery.

## Supplementary Materials

The following are available online at https://www.mdpi.com/1422-0067/21/7/2401/s1, Figure S1, Quality control of two different rVAR2 constructs and production batches by ELISA; Figure S2, Binding of different rVAR2 constructs and production batches to MyLa 2059 cells; Figure S3, rVAR2 CTC capture workflow; Figure S4, Addition of high rVAR2-SpyC concentrations to the beads results in bead aggregation using both a protein-free (PF) and BSA-based coupling buffer; Figure S5, Testing of mono- versus multi-biotinylated SpyC for bead binding and cancer cell retrieval; Table S1, Sensitivity assay using the direct rVAR2 method; Table S2, Cancer cell recoveries using the direct or indirect capture method; Table S3, Sensitivity assay using the indirect rVAR2 method.

## Abbreviations

BSA	Bovine serum albumin
CK	Cytokeratin
CS	Chondroitin sulfate
CTC	Circulating tumor cell
ctDNA	Circulating tumor DNA
CTG	CellTracker^TM^ Green
CTO	CellTracker^TM^ Orange
DBL1-ID2a	rVAR2 comprising Duffy-binding-like domain 1 to interdomain 2a
EDTA	Ethylenediaminetetraacetic acid (anticoagulant)
EMT	Epithelial to mesenchymal transition
EpCAM	Epithelial cell adhesion molecule
FBS	Fetal bovine serum
HSPGID1-ID2a	Heparan sulfate proteoglycanrVAR2 comprising interdomain 1 to interdomain 2a
IE	Infected erythrocyte
ofCS	Oncofetal chondroitin sulfate
DPBS	Dulbecco’s Phosphate Buffered Saline modified without Mg^2+^ and Ca^2+^
DPBS2	PBS with 2% added FBS
PF	Protein-free buffer
PFA	Paraformaldehyde
rVAR2	Recombinant VAR2CSA (DBL1-ID2a or ID1-ID2a)
SpyC	SpyCatcher
WBC	White blood cell
