# Supplementary material for: Optimization of rVAR2-Based Isolation of Cancer Cells in Blood for Building a Robust Assay for Clinical Detection of Circulating Tumor Cells
Source: Int J Mol Sci. 2020 Mar 31;21(7):2401. doi: 10.3390/ijms21072401 (PMC7178266; doi:10.3390/ijms21072401)
Supplement: Supplementary file 1 [file ijms-21-02401-s001.pdf]

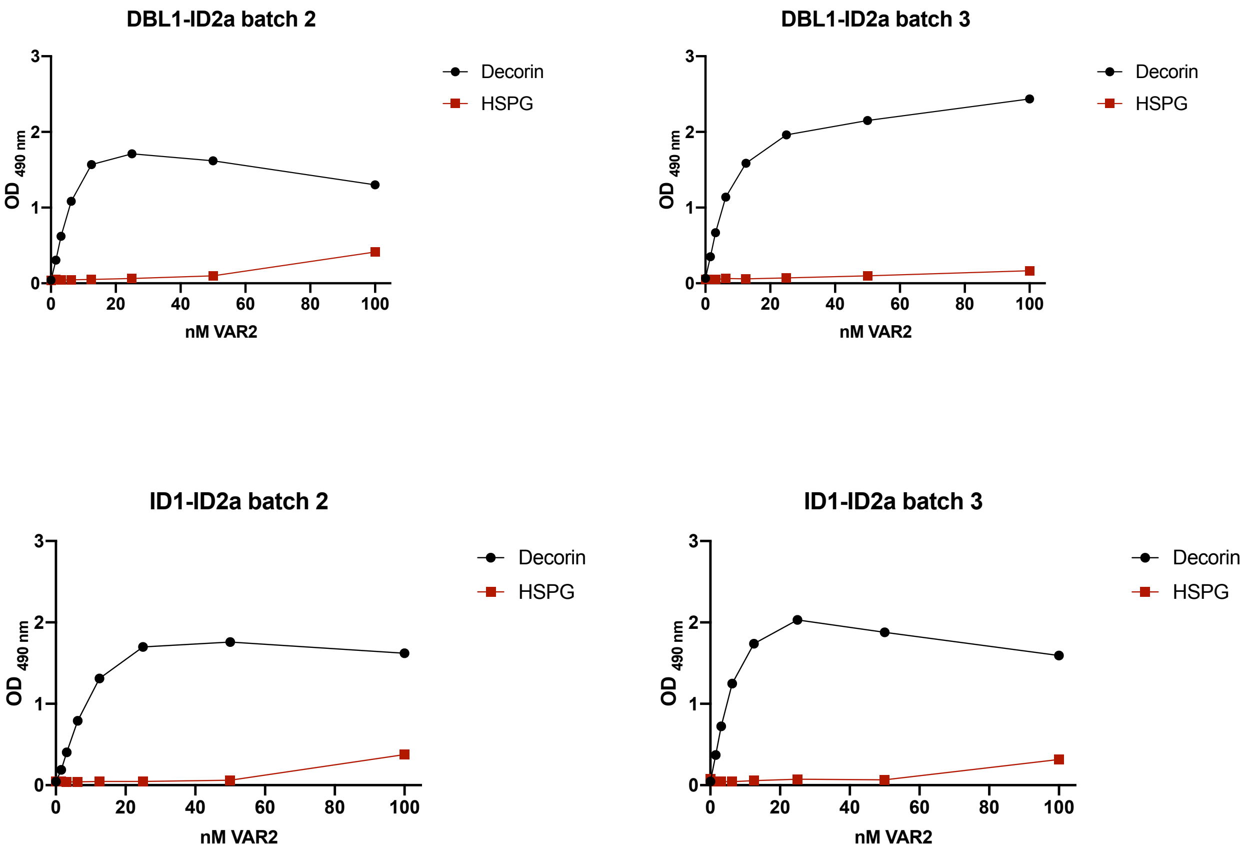

Figure S1. Quality control of two different rVAR2 constructs and production batches by ELISA. Two-fold dilutions (100–1.56 nM) of ID1-ID2a or DBL1-ID2a from different production batches were analyzed for binding to decorin (black) or heparan sulfate proteoglycan (HSPG, red) coated plates as measured by anti-His HRP reactivity.

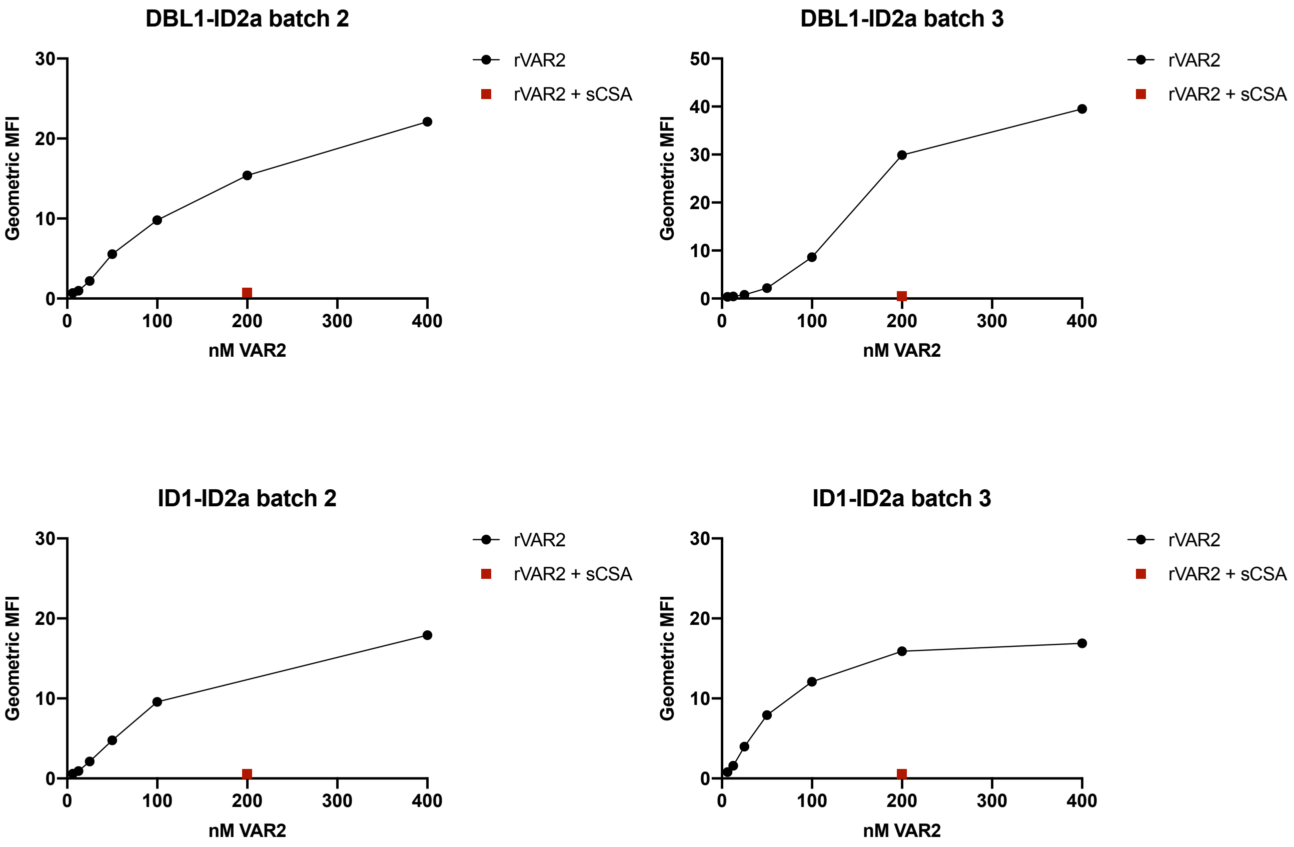

Figure S2. Binding of different rVAR2 constructs and production batches to MyLa 2059 cells. Two-fold dilutions (400–6.25 nM) of ID1-ID2a or DBL1-ID2a from different production batches were analyzed for binding to Myla 2059 cells as measured by anti-His Alexa 488 staining intensity by flow cytometry. Co-incubation with 400 µg/ml soluble CSA (sCSA, red) with rVAR2 and Myla 2059 cells completely inhibits rVAR2 binding.

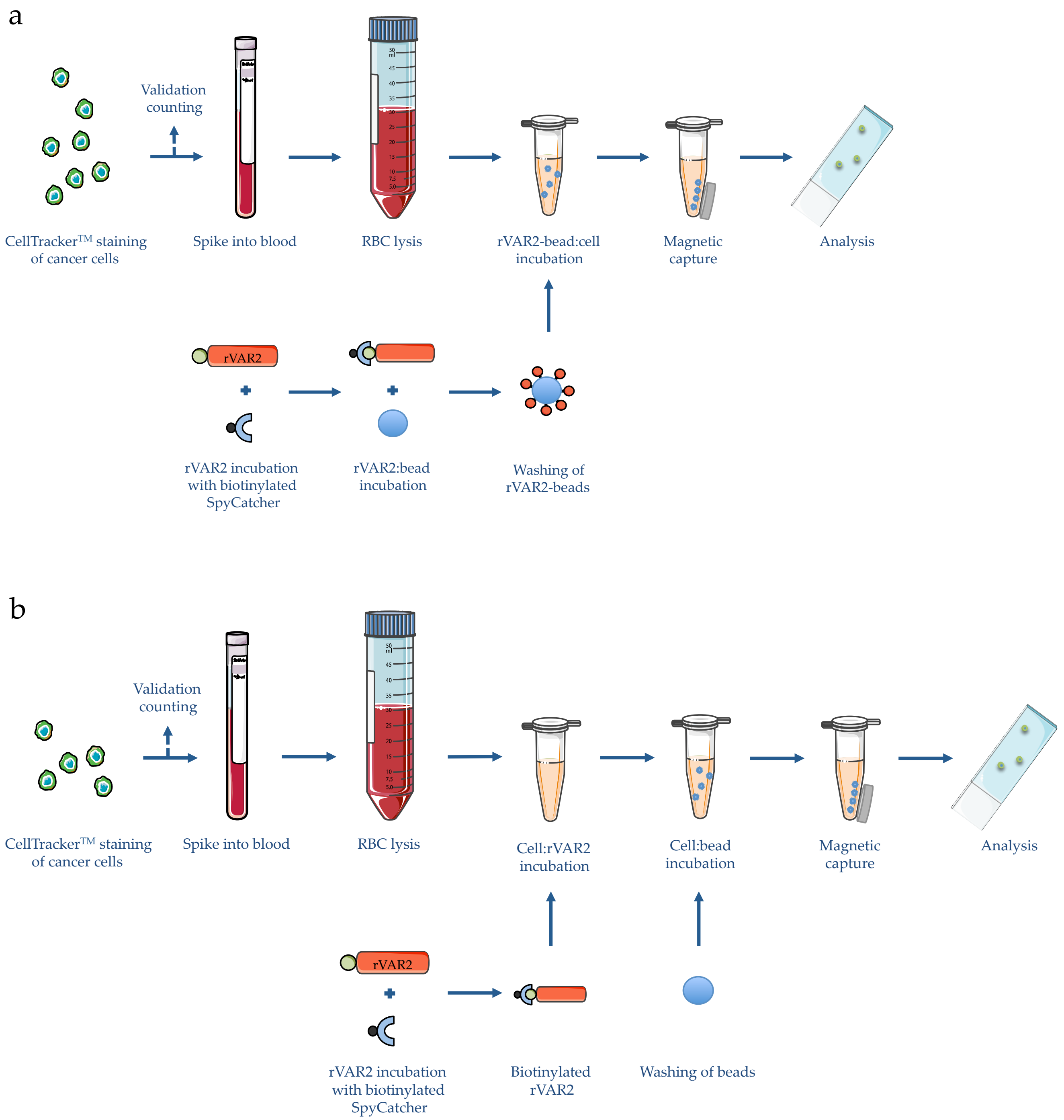

Figure S3. rVAR2 CTC capture workflow. (a) Direct approach. *Bottom:* Spytagged rVAR2 is biotinylated via binding to the biotinylated SpyCatcher and coated onto the Sera-Mag SpeedBeads Streptavidin-blocked Magnetic Particles. *Top:* CellTracker™-stained cancer cells are spiked into a 3 mL blood sample. In parallel, the amount of cancer cells is microscopically validated by 3 individual counts. Following RBC lysis, DPBS wash, and centrifugation the white blood cells and cancer cells are incubated with the rVAR2-coated beads. Bead-bound cells are then magnetically isolated, fixated, DAPI-stained, scanned and analyzed as described in Materials and Methods. (b) Indirect approach. *Bottom:* Spytagged rVAR2 is biotinylated via binding to the biotinylated SpyCatcher. *Top:* Biotinylated rVAR2 is incubated with the cell sample. Following centrifugation and wash to remove unbound rVAR2, the Sera-Mag SpeedBeads Streptavidin-blocked Magnetic Particles are added in a second incubation step to bind the rVAR2-coated cells. The remaining steps are identical to the direct approach. This figure was created using templates from Servier Medical Art website (https://smart.servier.com/).

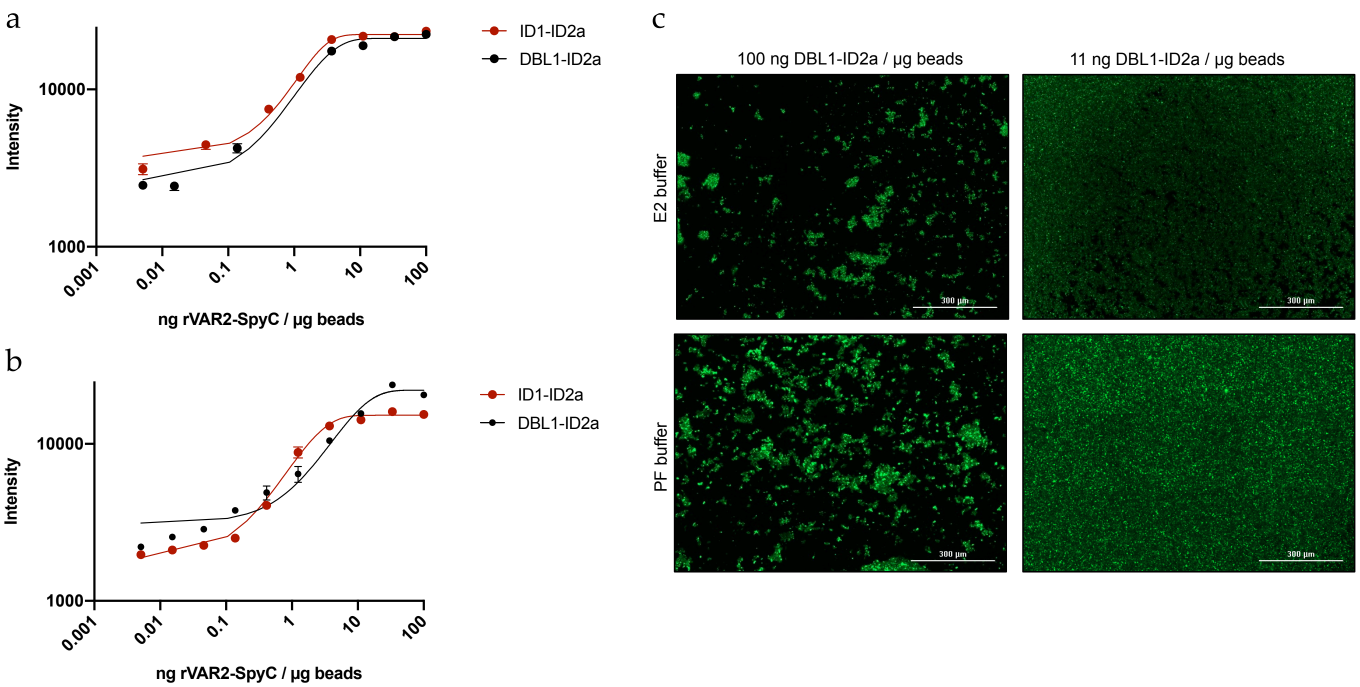

Figure S4. Addition of high rVAR2-SpyC concentrations to the beads results in bead aggregation using both a protein-free (PF) and BSA-based coupling buffer. Protein saturation of the beads was obtained around 10 ng rVAR2 per µg beads in both (a) BSA-based and (b) PF buffer, and further oversaturation did not increase rVAR2 density. (c) A protein to bead ratio of 100 ng/µg resulted in bead clumping independently of buffer type.

a

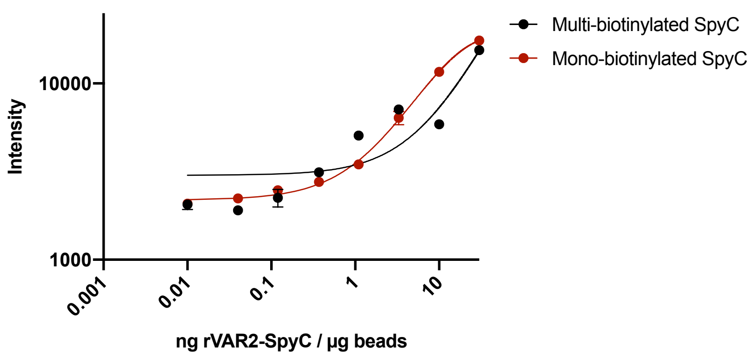

b

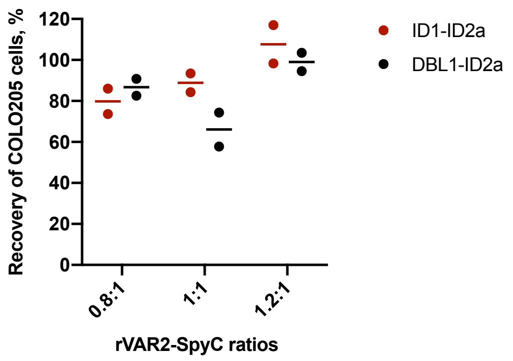

Figure S5. Testing of mono- versus multibiotinylated SpyC for bead binding and cancer cell retrieval (a) Multi- versus mono-biotinylated SpyCatcher coupled to DBL1-ID2a was tested for bead binding efficacy through rVAR2 density determination using Cytation™ 3 analysis. (b) Test of various rVAR2:SpyC ratios (0.8:1, 1:1 and 1.2:1) in cancer cell capture experiments. 100 CellTracker™ Green (CTG+) COLO205 cells were spiked into 1 mL blood and retrieved as described in materials and methods.

Table S1. Sensitivity assay using the direct rVAR2 method. Table specifying the recoveries of the direct capture using DBL1-ID2a on COLO205 cells and SW480 cells spiked into 3 mL of blood to assess assay sensitivity. [a] signifies the exact amounts of cells spiked in. [b] signifies mean of triplicate validation counts.

COLO205					SW480				
Spike-in	Recovered cells			Average recovery %	Spike-in	Recovered cells			Average recovery %
4[a]	3	2		63	4[a]	2			50
5[a]	5			100	5[a]	1			20
11[b]	9	6	7	67	7[a]	0			0
54[b]	49	34	44	78	14[b]	0	1	5	12
107[b]	80	66	62	65	42[b]	10	12	18	32
					110[b]	30	11	27	21

Table S2. Cancer cell recoveries using the direct or indirect capture method. Two sample Wilcoxon rank sum test was used to evaluate whether there was a statistical significant difference in the recovery of cancer cells using the two methods using a significance level of $P<0.05$. Data was analyzed by STATA 14.

	Mean recovery, %		
Cell line	Direct method	Indirect method	P-value
COLO205	69.4	98.6	0.0027
A549	56.4	98.9	0.0007
SW480	25.3	111.1	0.0054
SK-BR-3	12.3	47.2	0.0001
PC-3	49.1	61.2	0.0670

Table S3. Sensitivity assay using the indirect rVAR2 method. Table specifying the recoveries of the indirect capture using DBL1-ID2a on COLO205 cells and SW480 cells spiked into 3 mL of blood to assess assay sensitivity. [a] signifies the exact amounts of cells spiked in. [b] signifies mean of triplicate validation counts.

COLO205						SW480				
Spike-in	Recovered cells				Average recovery %	Spike-in	Recovered cells			Average recovery %
5[a]	3	1	2	5	55	3[a]	1			33
10[b]	7	9	8		80	4[a]	2			50
49[b]	45	47	54	59	105	5[a]	2			40
72[b]	70	65	75		97	9[b]	6	6	8	74
						46[b]	34	25	29	64
						84[b]	56	50	52	63
